# Comparison of short-term outcomes between robotic-assisted and laparoscopic gastrectomy guided by carbon nanoparticle suspension injection in gastric cancer

**DOI:** 10.1186/s12957-022-02755-3

**Published:** 2022-09-05

**Authors:** Zhiyan Li, Shichao Ai, Feng Wang, Liang Tao, Feng Sun, Peng Song, Xiaofei Shen, Qiongyuan Hu, Xianghui Li, Song Liu, Meng Wang, Wenxian Guan

**Affiliations:** Department of Gastrointestinal Surgery, Nanjing Drum Tower Hospital, The Affiliated Hospital of Nanjing University Medical School, 321 Zhongshan Rd., Nanjing, 210008 China

**Keywords:** Gastric cancer, Laparoscopic gastrectomy, Robotic-assisted gastrectomy, Carbon nanoparticle suspension injection

## Abstract

**Background:**

The clinical application of robotic-assisted gastrectomy remains controversial, especially as clinical studies of this operation navigated by carbon nanoparticle suspension injection (CNSI) have not been conducted. This study aims to assess the perioperative safety and efficacy of CNSI-guided robotic-assisted gastrectomy in patients with gastric cancer by focusing on short-term outcomes.

**Methods:**

A retrospective analysis of patients who underwent CNSI-guided laparoscopic or robotic-assisted gastrectomy with a pathological diagnosis of gastric cancer was conducted. Data on demographics, surgical management, clinical-pathological results and short-term outcomes were compared among the groups.

**Results:**

A total of 126 eligible patients were separated into the robotic-assisted gastrectomy (RAG) group (*n* = 16) and the laparoscopic gastrectomy (LG) group (*n* = 110) in total. The operation time of the RAG group is longer than the LG group (*p* = 0.0000). When it comes to perioperative and short-term complications, there exists no statistical difference between the two groups.

**Conclusion:**

The time required for CNSI-guided robotic-assisted gastrectomy is longer than that for CNSI-guided laparoscopic gastrectomy. CNSI-guided robotic-assisted gastrectomy is safe and effective.

## Introduction

Minimally invasive interventions and appropriate lymphadenectomy range are two major issues in gastric cancer surgery [1]. As an alternative to standard open gastrectomy, radical laparoscopic gastrectomy has been developed to decrease postoperative complications and hasten postoperative healing [2, 3]. Besides laparoscopic gastrectomy, robotic-assisted gastrectomy offers its distinct merits in improved dexterity and three-dimensional vision, which account for its recent popularity [1, 4].

On the other hand, lymph node acquisition is strongly linked to pathological staging and prognosis [5]. At present, gastrectomy with D2 lymphadenectomy is a standard surgical approach for advanced gastric carcinoma [6]. Harvesting lymph nodes (LNs) with lymphatic tracers is an ideal method for quality control. Among lymphatic tracers, carbon nanoparticle suspension injection is widely utilized for convenience, cheapness, and safety [7, 8]. The carbon nanoparticles with the size of 150 nm on average can be specifically uptaken by lymphatics and stain lymph nodes black for lymph node tracing during lymphadenectomy and postoperation lymph node harvesting [9].

Therefore, a combination of minimally invasive surgery and lymphatic tracers is a promising solution for precision surgery. In our institution, laparoscopic gastrectomy and robotic-assisted gastrectomy navigated by CNSI have been performed routinely. As reported, robotic-assisted gastrectomy is beneficial in increasing the number of retrieved LNs, decreasing intraoperative hemorrhage, lowering surgical complications, and shortening postoperative recovery time compared with laparoscopic or open surgery [10–12]. However, numerous previous clinical researches failed to identify the differences between laparoscopic and robotic-assisted gastrectomies in terms of outcomes [13–15]. Additionally, although CNSI does a favor to LN detection and better perioperative outcomes [16, 17], there is no clinical research evaluating the distinctions between these two surgical approaches with the assistance of CNSI. The current study is purposed to investigate the perioperative outcomes of laparoscopic gastrectomy and robotic-assisted gastrectomy in gastric cancer patients.

## Methods

### Patients

This retrospective study included patients who underwent radical robotic-assisted or laparoscopic gastrectomy at Nanjing Drum Tower Hospital, the Affiliated Hospital of Nanjing University Medical School, between July 2018 and February 2022. The robotic-assisted and laparoscopic gastrectomies shared the same indications, including a diagnosis of gastric cancer without LN involvement in the extraperigastric area or invasion of the serosal layer. Each patient received a thorough explanation of the surgery specifics. Whether to undergo robot-assisted or laparoscopic gastrectomy was at the discretion of the patient. All patients corresponded to the following inclusion criteria: (1) gastric adenocarcinoma was the pathology diagnosis of a preoperative endoscopic biopsy, (2) received robotic-assisted or laparoscopic gastrectomy guided by CNSI, (3) without endoscopic submucosal dissection before gastrectomy nor combined resection of other organs during operation, and (4) without distant metastasis. The exclusion criteria are as follows: (1) postoperative pathological diagnosis was not primary gastric adenocarcinoma, (2) conversion to open operation, (3) with preoperative chemotherapy, (4) American Society of Anesthesiology physical status score ≥ 4, and (5) pathological or clinical data was not complete. This study enrolled a total of 126 consecutive patients. All eligible cases were separated into two groups based on whether laparoscopic or robotic-assisted gastrectomy was used during the operation.

### Data collection

All data covered in this article, including demographic characteristics (age, gender, and BMI), preoperative data (preoperative histological type and tumor location), intraoperative events (surgical approach, operation time, and blood loss), postoperative pathological diagnosis, and short-term outcomes were all obtained from the clinical database.

### Administration of CNSI

**Fig. 1:**
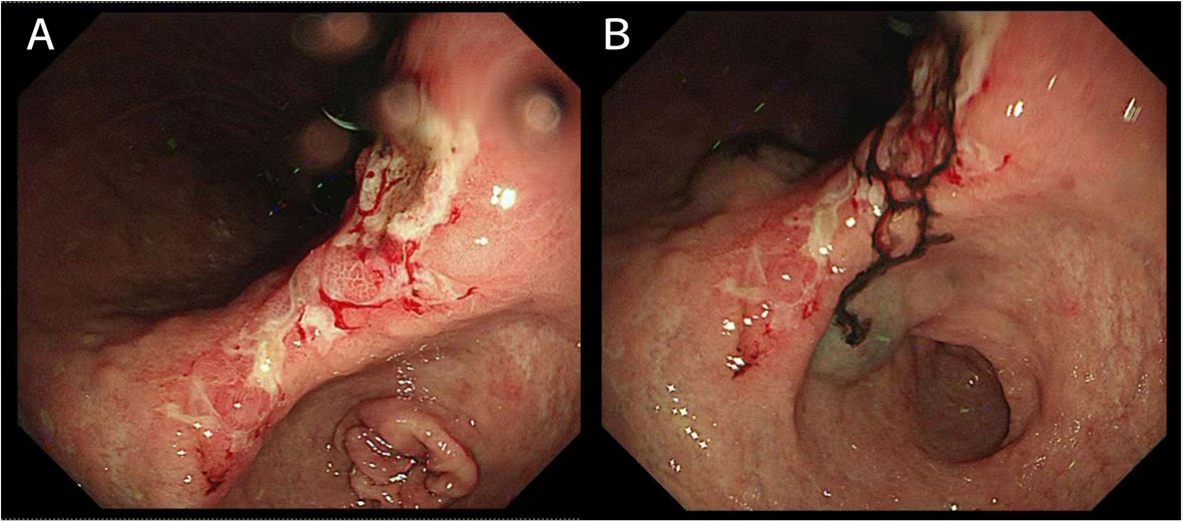
Preoperative peritumoral submucosal injection of CNSI. Endoscopic view. **A** Before injection. **B** After injection

Every patient enrolled received an endoscopic CNSI (Chongqing Lummy Pharmaceutical Co., Ltd., China) injection 1 day before the operation. After being diluted to a concentration of 25 mg/ml, the prepared CNSI solution powder was injected at the distal and proximal submucosa of the tumor, with 0.2 ml at each point (Fig. 1).

### Surgical approach

**Fig. 2:**
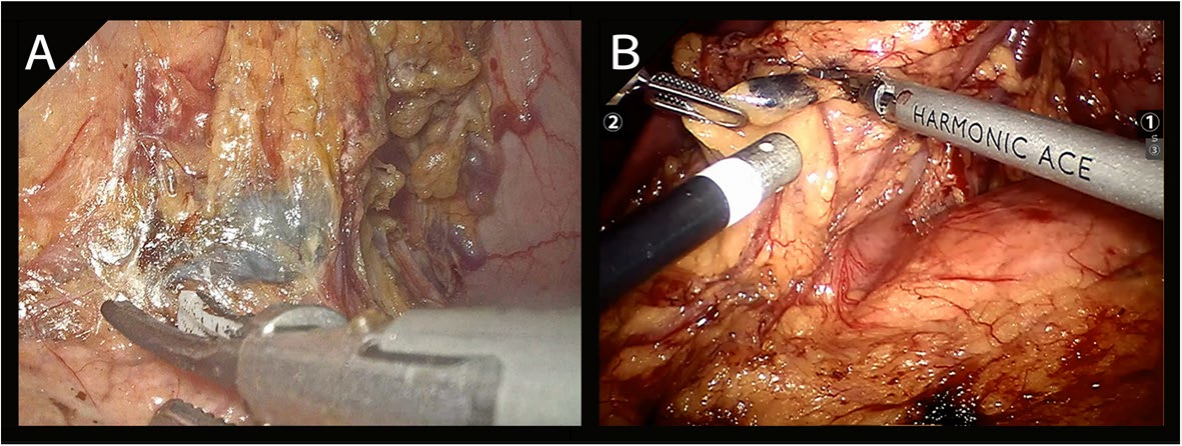
The intraoperative observations. Laparoscopic view. **A** CNSI-guided laparoscopic gastrectomy. **B** CNSI-guided robotic-assisted gastrectomy

T2–4 or N1–3 was the indication for radical distal gastrectomy, with a proximal resection margin of at least 3 cm for a localized tumor or at least 5 cm for an invasive tumor. If the proximal resection margin cannot fulfill the need for distal gastrectomy, radical total gastrectomy was the surgical approach for T2–4 or N1–3 tumors. For T1N0, over half of the stomach can be retained, and upper gastric tumors were indications for radical proximal gastrectomy. Roux-en-Y esophagojejunostomy reconstruction was applied in distal and total gastrectomy reconstruction, and double tract reconstruction, during which the remnant stomach was anastomosed with the distal jejunum in addition to performing Roux-en-Y esophagojejunostomy, was utilized for proximal gastrectomy reconstruction [18]. D2 lymphadenectomy was performed in all patients. Upper median incisions of about 5 cm were accepted to take out specimens for subsequent processing. The lymph node sorting approach followed the Japanese categorization [19]. The number of lymph nodes removed was determined by the postoperative pathology report. In sorted lymph node-like tissues, positive lymph nodes were defined as the presence of lymph nodes rather than fibrous connective tissue. The intraoperative observations of CNSI-guided laparoscopic and robotic-assisted gastrectomy are illustrated in Fig. 2.

### Statistical analysis

SPSS version 26.0 (SPSS Inc., Chicago, IL, USA) was used to analyze all statistics. All continuous variables were expressed as mean ± standard deviation and compared by either Student’s *t* test, Welch’s *t* test, or the Mann–Whitney *U* test, while the chi-square test or Fisher’s exact test was used to analyze the categorical data, which were displayed as frequency and percentage. There exists statistical significance when the *p*-value is less than 0.05.

### Ethics

The study was approved by the Ethics Committee of Nanjing Drum Tower Hospital, the Affiliated Hospital of Nanjing University Medical School. All participants enrolled provided written informed consent.

## Results

### Baseline characteristics

**Fig. 3:**
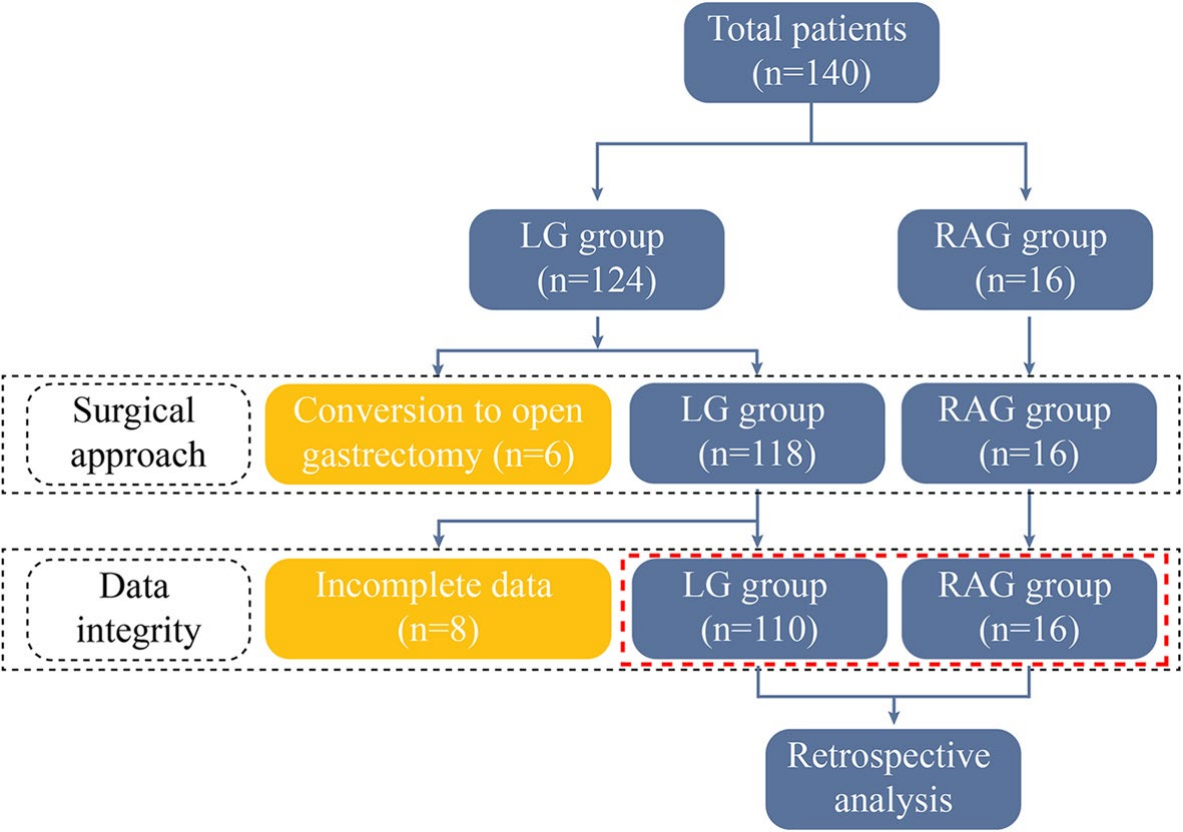
Study flow chart. A total of 140 consecutive cases underwent laparoscopic or robot-assisted gastrectomy. Six cases were excluded because of conversion to open gastrectomy, and 8 cases were excluded for incomplete clinical or pathological data. The remaining 126 cases were divided into two groups: the LG group (*n* = 110) and the RAG group (*n* = 16)

A total of 140 consecutive cases were performed laparoscopic or robot-assisted gastrectomy, as illustrated in Fig. 3. Due to conversion to open gastrectomy (6 cases) and incomplete clinical or pathological data (8 cases), fourteen cases were excluded. The remaining 126 cases were divided into two groups: the LG group (*n* = 110) and the RAG group (*n* = 16). The baseline characteristics of the LG and RAG groups did not differ statistically significantly, as shown in Table 1.

**Table 1:** Demographics and clinical characteristics between the LG and RAG groups

	LG (*n* = 110)	RAG (*n* = 16)	*p* value
Age (years)	56.90 ± 9.90	54.44 ± 12.07	0.1510
Gender (male, %)	77 (70.00%)	10 (62.50%)	0.5698
ASA-PS			0.1464
II	41 (37.27%)	3 (18.75%)	
III	69 (62.73%)	13 (81.25%)	
Comorbidities			
Cardiovascular	29 (26.36%)	7 (43.75%)	0.2533
Diabetes type 2	7 (6.36%)	1 (6.25%)	> 0.9999
Viral hepatitis	7 (6.36%)	2 (12.50%)	0.7106
Competing diseases			
Prostate cancer	1 (0.91%)	0 (0.00%)	> 0.9999
Esophageal cancer	0 (0.00%)	1 (6.25%)	0.1270
Thyroid cancer	0 (0.00%)	1 (6.25%)	0.1270
BMI (kg/m^2^)	23.08 ± 2.70	24.14 ± 3.68	0.1684
Tumor location			0.2348
Upper third	29	1	–
Middle third	25	5	–
Lower third	56	10	–
Histological type			0.7870
Well/moderate	38	6	–
Poor/undifferentiated	72	10	–

*ASA-PS* American Society of Anesthesiology Physical Status Classification

### Perioperative outcomes

Table 2 shows the perioperative outcomes of the two groups. Blood loss, type of resection, pT, pN, pStage, histological type, and postoperative hospital stay did not differ significantly. The operation time was visibly longer in the RAG group (*p* = 0.0000) than in the LG group. There were no intraoperative complications or postoperative death.

**Table 2:** Perioperative outcomes between the LG and RAG groups

	LG (*n* = 110)	RAG (*n* = 16)	*p* value
Operation time (min)	247.93 ± 54.56	324.38 ± 35.96	0.0000
Blood loss (ml)	112.93 ± 61.28	128.12 ± 51.54	0.7302
Type of resection			0.1171
Proximal gastrectomy	11	2	–
Distal gastrectomy	50	11	–
Total gastrectomy	49	3	–
Histological type			0.3869
Well/moderate	36	3	–
Poor/undifferentiated	74	13	–
pT stage			0.5305
pT1	59	8	–
pT2	23	2	–
pT3	24	6	–
pT4	4	0	–
pN stage			0.5586
pN0	70	8	
pN1	16	4	–
pN2	15	2	–
pN3	9	2	–
pSt stage			> 0.9999
I + II	91	13	–
III + IV	19	3	–
Number of retrieved LN (number)	29.32 ± 10.76	30.06 ± 9.14	0.6116
Postoperative hospital stay (days)	12.52 ± 4.62	12.38 ± 3.40	0.9054
Intraoperative complication	0	0	> 0.9999
Postoperative mortality	0	0	> 0.9999

TNM staging was based on the Japanese Classification of Gastric Carcinoma, 3rd English version

### Short-term outcomes

In the LG group, there were 21 cases of complications, including 1 fever, 1 intra-abdominal infection, 1 pulmonary infection, 2 lymphatic fistulas, 1 acute pancreatitis, 1 incision fat liquefaction, 7 gastropareses, 3 anastomotic bleedings, 1 bowel obstruction, 1 anastomosis leakage, 1 internal abdominal hernia, and 1 afferent loop obstruction, as indicated in Table 3. There were 3 cases in the RAG group, including 1 fever, 1 intra-abdominal infection, and 1 pancreatic fistula. The incidence of short-term complications was 19.09% in the LG group and 18.75% in the RAG group (*p* > 0.9999). A subgroup comparison of moderate (grades I and II) and serious (grades III and IV) complications between the groups revealed a similar prevalence.

**Table 3:** Short-term complications between the LG and RAG groups

	LG (*n* = 110)	RAG (*n* = 16)	*p* value
Overall (*n* (%))^a^	21 (19.09%)	3 (18.75%)	> 0.9999
Grade I or II (*n* (%))^a^	6 (5.46%)	2 (12.50%)	0.2684
Fever	1	1	–
Intra-abdominal infection	1	1	–
Pulmonary infection	1	0	–
Lymphatic fistula	2	0	–
Incision fat liquefaction	1	0	–
Grade III or IV (*n* (%))^a^	15 (13.64%)	1 (6.25%)	0.6912
Gastroparesis	7	0	–
Anastomotic bleeding	3	0	–
Bowel obstruction	1	0	–
Anastomosis leakage	1	0	–
Internal abdominal hernia	1	0	–
Afferent loop obstruction	1	0	–
Acute pancreatitis	1	0	–
Pancreatic fistula	0	1	–

^a^Clavien-Dindo’s classification of surgical complication

## Discussion

In recent years, robotic-assisted surgery is increasingly developed. Due to positive perioperative and postoperative outcomes as reported, robotic surgery has been introduced in many hospitals, including our institution. However, its application in gastric cancer remains controversial. Herein, we compared the outcomes of radical laparoscopic gastrectomy and robotic-assisted gastrectomy navigated by carbon nanoparticle suspension injection in patients with gastric cancer. The aim of the study was to evaluate the safety and efficiency of CNSI-guided robotic-assisted gastrectomy through statistical analysis, and the statistics demonstrated that there is no significant difference in these two surgical approaches apart from longer operation time in the robotic-assisted group.

The operation time, recorded from patients entering the operating room to abdominal closure, was counted and analyzed. Several previous researches concluded that robotic-assisted gastrectomy spent more time than laparoscopic gastrectomy [12, 20, 21]. Analogously, robot-assisted gastrectomy took on average over 75 min longer than laparoscopic gastrectomy in our study. One probable explanation is that setup and docking of the robotic arms take more time, as well as the time required for arm change during clipping [22]. Interference from camera motion or the maladaptive surgical field might be another factor [10]. However, it demonstrated that garbage time could be shortened significantly with increased experience [23]. The drawbacks of robot-assisted gastrectomy can be mitigated by this. Additionally, although the cases included in this article were performed by surgeons with extensive experience in laparoscopic and robotic-assisted surgeries, the selected CNSI-guided robotic-assisted gastrectomies were among the first few cases. The insufficient adaptation time and required learning curve possibly slightly lengthened the operation time due to the differences caused by the application of CNSI. According to previous experience, the difference in operation time will narrow with the accumulation of operation experience [22]. As other reports mentioned, robot-assisted surgery spent a similar, even shorter time than traditional laparoscopic surgery in lobectomy or hysterectomy [24, 25], indicating that the complexity and the specific steps of the surgery possibly account for the operation time difference between robotic-assisted and laparoscopic surgeries. Nevertheless, there is evidence that a longer operation time is not closely associated with a poor prognosis [26], which was also illustrated in our data.

The appropriate lymphadenectomy is one of the topics of gastrectomy since the precise pathological staging of gastric cancer, follow-up therapy, and patient survival are closely related to perigastric lymph node dissection [27]. Following the proposal of the Japanese Regulations on the Management of Gastric Cancer, at least 15 lymph nodes should be dissected [18]. If more than 30 lymph nodes are retrieved for evaluation, the postoperative N staging will be more accurate [28]. Therefore, CNSI is usually used to assist in the dissection of lymph nodes. Whether in the LG or RAG group, the average number of detected lymph nodes was close to 30 in our research, which was far exceeding the number specified in the guidelines. Although the injecting methods of CNSI are divided into submucosal and subserosal [9, 16], we adopted submucosal injections 1 day before surgery since a waiting time of over 6 h seemed to be more appropriate for imaging [17]. Whether in robotic-assisted or laparoscopic gastrectomy, the lymph nodes in the surgical field were all stained black, which was more convenient for the identification and detection of lymph nodes for precise N staging.

One referred advantage of robotic-assisted gastrectomy is more retrieved lymph nodes than laparoscopic gastrectomy during operation [10, 13], partly attributed to the excellent three-dimensional visualization. The advantage is even magnified when exact dissection is required along the main arteries of the abdomen [11, 13]. In contrast to previous studies, our investigation revealed no significant variation in the number of lymph nodes extracted. The usage of CNSI probably accounted for the consequence. As reported before, CNSI improved the quantity and accuracy of lymph nodes collected significantly due to their excellent lymphatic system targeting and retention capacities [9, 16]. Thus, it has been widely applied in lymph node detection. Therefore, the combination of CNSI and radical laparoscopic gastrectomy might make up for the shortage of the narrow visual field of endoscopic surgery in lymphadenectomy and leave little room for improvement through robotic-assisted surgery.

Apart from the factors discussed above, there was no remarkable distinction between the LG and RAG groups in both perioperative and short-term outcomes. Gastrectomy with the assistance of a robot was not linked to an increased risk of perioperative complications, which is consistent with earlier research [12, 13]. Similar postoperative hospital stays indicated a similar recovery process in both groups. Neither intraoperative complication nor postoperative death happened, proving that the safety of these surgical methods was similar. What is more, the incidence and severity of short-term complications were also comparable in both groups. Our findings identified the safety of robotic-assisted gastrectomy guided by CNSI.

Thence, we assume that the safety and efficiency of CNSI-guided robotic-assisted gastrectomy are guaranteed. Besides, certain advantages of robotic-assisted surgery, including the shorter learning curve for surgeons with little prior expertise with minimally invasive surgery, are tough to be revealed in the data directly. However, we did not find any unique advantages of robotic-assisted surgery based on our data. We believe that both laparoscopic and robotic-assisted gastrectomies guided by CNSI are superior options until the merits of robotic-assisted surgery are clearly demonstrated.

There are various limitations in our research. First, this is a single-center research with a limited sample, and only a small number of patients underwent robotic-assisted gastrectomy because the surgical procedure was conducted in the last few years, which might lead to selection bias. Second, long-term outcomes require a longer follow-up time. However, RAG under the guidance of CNSI has not been carried out in our center until recent years. Therefore, long-term outcomes were not included for their inaccuracy. Third, since few patients received traditional CNSI-guided open gastrectomy, we failed to compare LG and RAG with open gastrectomy to assess their variances. Further larger multicenter randomized studies are anticipated to provide more information and validate our findings.

## Conclusion

CNSI-guided robotic-assisted gastrectomy takes more time than CNSI-guided laparoscopic gastrectomy. When it comes to perioperative and short-term outcomes, CNSI-guided robotic-assisted gastrectomy is safe and effective.

## Data Availability

The datasets used and/or analyzed during the current study are available from the corresponding authors upon reasonable request.

## Abbreviations

CNSI: Carbon nanoparticle suspension injection
RAG: Robotic-assisted gastrectomy
LG: Laparoscopic gastrectomy
LN: Lymph node

## References

[1] Smyth EC (2020). Gastric cancer. Lancet.

[2] Kinoshita T (2019). Long-term outcomes of laparoscopic versus open surgery for clinical stage II/III ggastric cancer: a multicenter cohort study in Japan (LOC-A Study). Ann Surg.

[3] Yu J (2019). Effect of laparoscopic vs open distal gastrectomy on 3-year disease-free survival in patients with locally advanced gastric cancer: the CLASS-01 Randomized Clinical Trial. JAMA.

[4] Zhao EH, Ling TL, Cao H (2016). Current status of surgical treatment of gastric cancer in the era of minimally invasive surgery in China: opportunity and challenge. Int J Surg.

[5] Lu X (2021). The short-term and long-term outcomes of indocyanine green tracer-guided laparoscopic radical gastrectomy in patients with gastric cancer. World J Surg Oncol.

[6] Martín-Richard M (2020). SEOM clinical guideline for the diagnosis and treatment of gastric cancer (GC) and gastroesophageal junction adenocarcinoma (GEJA) (2019). Clin Transl Oncol.

[7] Hagiwara A (1992). Lymph nodal vital staining with newer carbon particle suspensions compared with India ink: experimental and clinical observations. Lymphology.

[8] Chen H (2014). Application of subserosal injection of carbon nanoparticles via infusion needle to label lymph nodes in laparoscopic radical gastrectomy. Zhonghua Wei Chang Wai Ke Za Zhi.

[9] Li Z (2016). Clinical study of harvesting lymph nodes with carbon nanoparticles in advanced gastric cancer: a prospective randomized trial. World J Surg Oncol.

[10] Cianchi F (2016). Robotic vs laparoscopic distal gastrectomy with D2 lymphadenectomy for gastric cancer: a retrospective comparative mono-institutional study. BMC Surg.

[11] Caruso S (2011). Open vs robot-assisted laparoscopic gastric resection with D2 lymph node dissection for adenocarcinoma: a case-control study. Int J Med Robot.

[12] Parisi A (2017). Minimally invasive surgery for gastric cancer: a comparison between robotic, laparoscopic and open surgery. World J Gastroenterol.

[13] Junfeng Z (2014). Robotic gastrectomy versus laparoscopic gastrectomy for gastric cancer: comparison of surgical performance and short-term outcomes. Surg Endosc.

[14] Obama K (2018). Long-term oncologic outcomes of robotic gastrectomy for gastric cancer compared with laparoscopic gastrectomy. Gastric Cancer.

[15] Liu HB (2018). Robotic versus conventional laparoscopic gastrectomy for gastric cancer: a retrospective cohort study. Int J Surg.

[16] Feng Y (2021). Value of preoperative gastroscopic carbon nanoparticles labeling in patients undergoing laparoscopic radical gastric cancer surgery. Surg Oncol.

[17] Zhao K (2022). Role of carbon nanotracers in lymph node dissection of advanced gastric cancer and the selection of preoperative labeling time. World J Clin Cases.

[18] Japanese Gastric Cancer Association (2017). Japanese gastric cancer treatment guidelines 2014 (ver. 4). Gastric Cancer.

[19] Japanese classification of gastric carcinoma: 3rd English edition. Gastric Cancer 2011 14(2):101–12. 10.1007/s10120-011-0041-5 21573743

[20] Gao Y (2019). Comparison of robotic- and laparoscopic-assisted gastrectomy in advanced gastric cancer: updated short- and long-term results. Surg Endosc.

[21] Okumura N (2016). Robotic gastrectomy for elderly gastric cancer patients: comparisons with robotic gastrectomy in younger patients and laparoscopic gastrectomy in the elderly. Gastric Cancer.

[22] Park JY (2015). Robot-assisted gastrectomy for early gastric cancer: is it beneficial in viscerally obese patients compared to laparoscopic gastrectomy?. World J Surg.

[23] Song J (2009). Robot-assisted gastrectomy with lymph node dissection for gastric cancer: lessons learned from an initial 100 consecutive procedures. Ann Surg.

[24] Huang J (2021). Comparison of perioperative outcomes of robotic-assisted versus video-assisted thoracoscopic right upper lobectomy in non-small cell lung cancer. Transl Lung Cancer Res.

[25] Lönnerfors C, Reynisson P, Persson J (2015). A randomized trial comparing vaginal and laparoscopic hysterectomy vs robot-assisted hysterectomy. J Minim Invasive Gynecol.

[26] Hwang SH (2009). Risk factors for operative complications in elderly patients during laparoscopy-assisted gastrectomy. J Am Coll Surg.

[27] Deng J (2017). Increasing the number of examined lymph nodes is a prerequisite for improvement in the accurate evaluation of overall survival of node-negative gastric cancer patients. Ann Surg Oncol.

[28] Ichikura T (2003). Minimum number of lymph nodes that should be examined for the International Union Against Cancer/American Joint Committee on Cancer TNM classification of gastric carcinoma. World J Surg.

